# Amphetamine enhances endurance by increasing heat dissipation

**DOI:** 10.14814/phy2.12955

**Published:** 2016-09-07

**Authors:** Ekaterina Morozova, Yeonjoo Yoo, Abolhassan Behrouzvaziri, Maria Zaretskaia, Daniel Rusyniak, Dmitry Zaretsky, Yaroslav Molkov

**Affiliations:** ^1^ Department of Physics Indiana University Bloomington Indiana; ^2^ Department of Mathematical Sciences Indiana University – Purdue University Indianapolis Indiana; ^3^ Department of Emergency Medicine Indiana University School of Medicine Indianapolis Indiana; ^4^ Department of Mathematics and Statistics Georgia State University Georgia

**Keywords:** Exercise, modeling, stimulants, thermoregulation

## Abstract

Athletes use amphetamines to improve their performance through largely unknown mechanisms. Considering that body temperature is one of the major determinants of exhaustion during exercise, we investigated the influence of amphetamine on the thermoregulation. To explore this, we measured core body temperature and oxygen consumption of control and amphetamine‐trea ted rats running on a treadmill with an incrementally increasing load (both speed and incline). Experimental results showed that rats treated with amphetamine (2 mg/kg) were able to run significantly longer than control rats. Due to a progressively increasing workload, which was matched by oxygen consumption, the control group exhibited a steady increase in the body temperature. The administration of amphetamine slowed down the temperature rise (thus decreasing core body temperature) in the beginning of the run without affecting oxygen consumption. In contrast, a lower dose of amphetamine (1 mg/kg) had no effect on measured parameters. Using a mathematical model describing temperature dynamics in two compartments (the core and the muscles), we were able to infer what physiological parameters were affected by amphetamine. Modeling revealed that amphetamine administration increases heat dissipation in the core. Furthermore, the model predicted that the muscle temperature at the end of the run in the amphetamine‐treated group was significantly higher than in the control group. Therefore, we conclude that amphetamine may mask or delay fatigue by slowing down exercise‐induced core body temperature growth by increasing heat dissipation. However, this affects the integrity of thermoregulatory system and may result in potentially dangerous overheating of the muscles.

## Introduction

In many conditions exhaustion may serve as an important safety mechanism keeping the organism from irreversible damage caused by intense exercise (Noakes 2012). It was previously shown that low to moderate doses of amphetamine increase the time until exhaustion in exercising rats (Wyndham et al. 1971; Gerald 1978). Although amphetamine usage is prohibited during competitions, it may be used in some situations to improve performance by delaying exhaustion (WADA, 2012). However, the mechanism by which amphetamine increases the time to exhaustion is unknown.

High body temperature is one of the strongest exhaustion signals (Walters et al., 2000). During exercise, the temperature of the muscles as well as the core body temperature is elevated as a consequence of increased heat production in the muscles. To limit the temperature growth, regulatory heat dissipation mechanisms, for example, vasodilatation and evaporative cooling through saliva spreading in rodents or sweating in humans, are engaged to help remove heat during physical exercise (Young and Dawson 1982; Horowitz et al. 1983). The balance between heat production and heat dissipation is crucial for keeping the temperature of different compartments of the body in a safe range.

Amphetamine is known to affect the thermoregulatory system (Borbely et al. 1974) by altering both heat production and heat dissipation. It has been previously shown that amphetamine increases the temperature at which exhaustion occurs at a high ambient temperature, producing a risk of developing exertional heat stroke (Zaretsky et al. 2014). Production of large amounts of heat by the muscles during exercise results in muscle tissue temperature being higher than core temperature. Muscle tissue itself can be damaged if its temperature becomes too high (Kregel 2002). Damage to the myocytes releases the content of the cells into the circulation. Misbalance of electrolytes can lead to cardiac arrhythmias, while a release of myoglobin may cause renal failure (Lima et al. 2008). Unfortunately, thermal or biochemical damage to the myocytes is not usually detected until post exercise. Therefore, any alterations in the thermoregulatory system that lead to higher muscle temperatures can be extremely dangerous.

In this study we aimed to find how amphetamine affects the mechanisms of exhaustion in exercising rats. To do so, we collected experimental data on heat production and the core temperature in rats running on a treadmill, and calculated the unobserved parameters, such as muscle temperature and heat dissipation, using our previously published mathematical model (Yoo et al. 2015). This approach helped us to identify a novel and counterintuitive mechanism underlying ergogenic effect of amphetamine, as well as provided new arguments on potential danger of using psychostimulants to improve performance during exercise.

## Methods

### Animals and experimental design

Male adult Sprague Dawley rats (300 ± 20 g, Harlan, Indianapolis, IN) were maintained in a 12‐h light/dark cycle (lights on between 7 am and 7 pm) with free access to food and water. All experiments were conducted between 10:00 am and 4:00 pm at room temperature (24°C). All procedures were approved by the Indiana University Animal Care and Use Committee.

### Drugs

d‐Amphetamine was obtained from Sigma‐Aldrich (St. Louis, MO) and dissolved in sterile saline (0.9%), so that the volume of injection would be 1 mL/kg of body weight. Injections were performed intraperitoneally.

### Surgical preparation

Animals were anesthetized with 1.5–2% isoflurane in oxygen. For the measurement of core body temperature, TA‐F40 telemetric transmitters (DSI, St. Paul, MN) were implanted intraperitoneally (i.p.) via a 2‐cm‐long longitudinal medial skin incision and muscular wall incision at the linea alba. Following insertion of the transmitter into the abdominal cavity, rats were returned to their cages for at least 1 week before treadmill familiarization.

### Treadmill familiarization

Prior to experiments, rats were familiarized to running on a treadmill (Columbus Instruments, Columbus, OH) for 14 days. Rats were run on the treadmill for 5 min/day with both the slope and speeds increasing daily: on day 1 rats were run at 5° incline at a maximum speed of 10 m/min and by day 15 at an incline of 20° and a maximum speed of 26 m/min. Familiarization customizes rats to the running on a treadmill, but does not induce training adaptations (Lambert and Noakes 1989). Mild electric stimulus at the back of the treadmill chamber promoted learning of the running behavior. Animals that were unable to run by the end of the familiarization sessions were eliminated from the study.

### Measurements of oxygen consumption and heat production

Measurements were obtained using an indirect open circuit calorimetric system (Oxymax, Columbus Instruments, Columbus, OH). Gas analyzer calibrations were conducted before testing using standardized gas mixtures (Praxair, Danbury, CT). After the lane of treadmill was plugged with a plate connected to a gas analyzer, a period of equilibration (approximately 5 min) was needed before measurements were started. After that, O_2_ consumption (*V*O_2_) and CO_2_ production (*V*CO_2_) were recorded once every minute, and data were expressed relative to body weight.

### Experimental design

**Figure 1:**
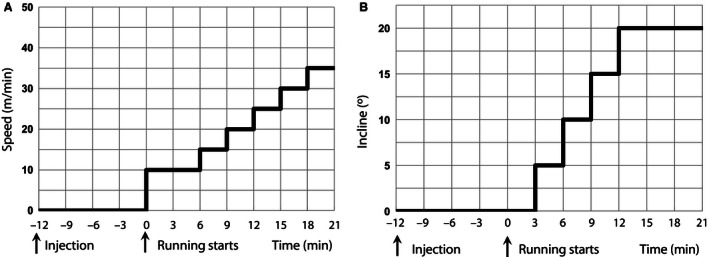
The experimental protocol. (A) Speed and (B) incline of the treadmill. Rats were injected with amphetamine or saline 12 min before the treadmill belt was activated.

Two groups of rats were used in the study (*N* = 6 in each group): one receiving 1 mg/kg of amphetamine and second receiving 2 mg/kg of amphetamine. Within each group a repeated trials crossover design was utilized. Rats that had been appropriately familiarized to running were randomized to receive in the first trial either the dose of amphetamine or equivalent volume of saline. In the next trial (at least 2 days after the first experiment) animals were subjected to the second experiment in which saline animals received amphetamine and vice versa. On the day of experiment rats were brought to the experimental room and allowed to adapt to the experimental condition. Telemetric recording of body temperature was initiated. After at least 30 min of baseline recording, the animal was administered with amphetamine or saline intraperitoneally according to the protocol. Immediately after the injection, animals were placed on a belt of the treadmill, and gas analysis was initiated. After 12 min, the treadmill was activated with the intensity of exercise increased every 3 min according to the experimental protocol (Fig. 1). Exhaustion was defined as the point at which a rat stopped keeping up with the speed of the treadmill and received three consecutive electric shocks. The core temperature was recorded once every minute by telemetric system (ART Dataquest, DSI, St. Paul, MN). The oxygen consumption (*V*O_2_) was measured throughout the experiment.

### Data analysis and statistical procedures

We calculated the average and the standard deviations of the core body temperatures and *V*O_2_ for all groups. Considering that the dose of 1 mg/kg of amphetamine did not affect body temperature with statistical significance, for modeling purposes we only used data for the control group (injected with saline) and amphetamine group which received 2 mg/kg of amphetamine. In order to obtain consistent datasets, we discarded data points starting when at least one of the rats in each group dropped out. Thus, the experimental data from *t = −*12 min to *t = *13 min for saline (*n* = 6), and from *t* = −12 min to *t = *15 min for amphetamine (*n* = 6) was used for estimating model parameters. So, we used ${\left\{T_{k}\left(t\right)\right\}}_{t=-12}^{N}$ as the set of data points for *k*th group (where *k* = saline or Amph) and *N = *13 or 15 for saline and Amph, respectively. The initial *V*O_2_ level was measured after the calibration time of the instrument. The level was steady before the running started. Accordingly, we considered O_2_ consumption constant from −12 min to 0 min for each group and equal to the mean value of *V*O_2_ at *t = *0 min. The experimental data used for the model calibration are shown in Figure 3.

Between‐group comparison of heat dissipation and heat production was performed using *z*‐test. Statistical significance was defined as a *P *< 0.05. Values are presented as mean ± SE.

### Model design

In order to quantify heat generation and heat loss in all groups, we adapted a mathematical model describing the temperature changes in two compartments, representing the core and the muscles (Yoo et al. 2015). In the model, the heat production parameter in the core, *P*_c_, is defined as total heat generated in the core body per time unit divided by the heat capacity of the core. Similarly, the heat production in the muscles, *P*_m_ is extra heat generated in the muscles by physical activity per time unit divided by heat capacity of the muscles. Both parameters have units of °C/min. The muscles exchange heat with the core proportional to the heat transfer coefficient *η* and the difference between temperatures of two compartments $\left(T_{c}-T_{m}\right)$. The value of *η* was fixed at 0.125 min^−1^ based on Yoo et al. (2015). Heat exchange between the core and environment is proportional to another heat transfer coefficient *η*_a_ and the difference of temperatures between the core and environment, $\left(T_{c}-T_{a}\right)$. Therefore, changes in the core and the muscle temperatures are described by a system of two first‐order differential equations.

(1) $$\begin{array}{cc} & \frac{\text{d}T_{c}}{\;\text{dt}}=P_{c}-\eta \left(T_{c}-T_{m}\right)-\eta_{a}\left(T_{c}-T_{a}\right);\\ & \frac{dT_{m}}{\text{dt}}=P_{m}-\eta \left(T_{m}-T_{c}\right). \end{array}$$

As noted, the heat production and the heat dissipation parameters in this model have units of °C/min. However, they can be easily converted into units of power (J/min) by multiplying by the heat capacity (in J/°C) of the corresponding compartment (Gordon 1990).

In accordance with the experimental data (Gavini et al. 2014), we assumed that at the beginning of the experiment (prior to the run) the temperature of the muscles was equal to the temperature of the core. Specifically, *T*_c_ and *T*_m_ are the same at *t* = −12 min when each rat was just placed on a treadmill after an injection: the initial conditions are *T*_m_(−12) = *T*_c_(−12) = *T*_0_. Initial temperature for each group was calculated as the averaged core body temperature from *t *= −15 min to *t *= −12 min.

Almost all the energy generated in the body is due to oxidation. Total energy production in the body is proportional to the amount of consumed oxygen, $VO_{2}$ (L/kg/min). Some portion of the energy is spent for the mechanical work (MW, cal/kg/min), and the rest is transformed to the heat. Accordingly, after denoting the coefficient of proportionality between total energy production and oxygen consumption by *α*, for the total heat production we have:

$$m_{m}P_{m}+m_{c}P_{c}=\left(m_{m}+m_{c}\right)\left(\alpha \cdot VO_{2}\left(t\right)-MW\right).$$

Here, *m*_m_ and *m*_c_ are heat capacities of the muscles and the core, respectively, and *m*_m_ + *m*_c_ is a total heat capacity of a rat. Taking into account that the skeletal muscle mass of a rat constitutes approximately 45–50% of its body weight (Franco et al. 2011) with the mass of a skeleton constituting only about 3%, we set *m*_m_ = *m*_c_ in the model. Accordingly, the above equation reduces to the form of:

(2) $$P_{m}+P_{c}=2\left(\alpha \cdot VO_{2}\left(t\right)-MW\right).$$

The coefficient *α* can be calculated as calorific value (CV) divided by the specific heat of the body. The latter for a rat is approximately 0.8 kcal/(kg·°C) (Gordon 1990). CV is calculated as:

CV=3.815+1.232RER(kcal/L),

where RER stands for the respiratory exchange ratio, which is the ratio of the carbon dioxide production (*V*CO_2_) to the oxygen consumption (*V*O_2_) (Lusk 1928).

The mechanical work consists in climbing the treadmill at the given *speed* (m/min) and *incline* (°) (see Fig. 1) and can be estimated as MW = *g*·*speed*·tan(*incline*)/4.184 in cal/kg/min, where *g *=* *9.8 m/sec^2^ is gravitational acceleration.

### Model parameter estimation

Since *V*O_2_ and *V*CO_2_ were measured, once we determine *P*_c_, then *P*_m_ can be calculated using equation (2). Accordingly, equation (1) has two undetermined independent parameters, *P*_c_ and *η*_a_. We used Bayesian approach to find statistical distributions for these parameters. Specifically, for each group *k*, measured core temperature values ${\left\{T_{k}\left(t\right)\right\}}_{t=-12}^{N}$ were considered independent normal random variables with the mean values *T*_c_(*η*_a_, *P*_c_, *t*) (solution of eq. [1]), and standard deviations *σ*_k_(*t*) calculated from experimental data. Based on this assumption, the likelihood (conditional joint probability density function) was calculated:

(3) $$\begin{array}{cc} & L({\left\{T_{k}\left(t\right)\right\}}_{t=-12}^{N}|\eta_{a},P_{c})\\ & ∼\exp\left\{-\frac{1}{2}\sum_{t=0}^{N}\frac{{\left(T_{k}\left(t\right)-T_{c}\left(\eta_{a},P_{c},t\right)\right)}^{2}}{\sigma_{k}^{2}\left(t\right)}\right\} \end{array},$$

where *k *= saline or Amph, and *N* = 13 and 15 for saline and Amph, respectively. The likelihood (equation 3) was sampled using Markov Chain Monte Carlo approach by Metropolis–Hastings algorithm (Mukhin et al. 2006; Loskutov et al. 2008; Molkov et al. 2011, 2012, 2014; Robert et al.).

### Glossary

**Table:** 

*T*_c_	Core body temperature in the model (°C)
$\frac{dT_{c}}{\text{dt}}$	Rate of change of the core temperature with respect to time $\left({}^{\circ }C/min\right)$
*T*_m_	Temperature of muscles in the model (°C)
$\frac{dT_{m}}{\text{dt}}$	Rate of change of the temperature of the muscles with respect to time $\left({}^{\circ }C/min\right)$
*T*_0_	Initial temperature of the core body and the muscles before running (°C)
*T*_*a*_	Ambient temperature (°C)
*P*_*c*_	Heat produced by the core per minute (°C/min)
*P*_*m*_	Heat produced by the muscles due to exertion per minute (°C/min)
*t*	Time (min)
*m*_c_	Mass of core body (kg)
*m*_m_	Mass of skeletal muscles (kg)
*CV*	Calorific value
*V*O_2_	Oxygen consumption (*mL*/[*kg*·min])
*V*O_2_max	Maximal observed oxygen consumption (*mL*/[*kg*·min])
*V*CO_2_	Carbon dioxide consumption (*mL*/[*kg*·min])
RER	Respiratory exchange ratio
*η*_a_	Heat dissipation coefficient in the core ${\left(min\right)}^{-1}$
*η*	Heat transfer coefficient between muscles and core ${\left(min\right)}^{-1}$
${\left\{T_{k}\left(t\right)\right\}}_{t=-12}^{N}$	A set of average core body temperature time‐series in group *k*,* k*= Saline or Amph, and *N = *13 min or 15 min, respectively (°C)
$\sigma_{k}^{2}\left(t\right)$	Sample variance of core body temperature at time *t* (*min*) in group *k* (*k*=Saline or Amph) (°C)

## Results

### Experimental data

**Figure 2:**
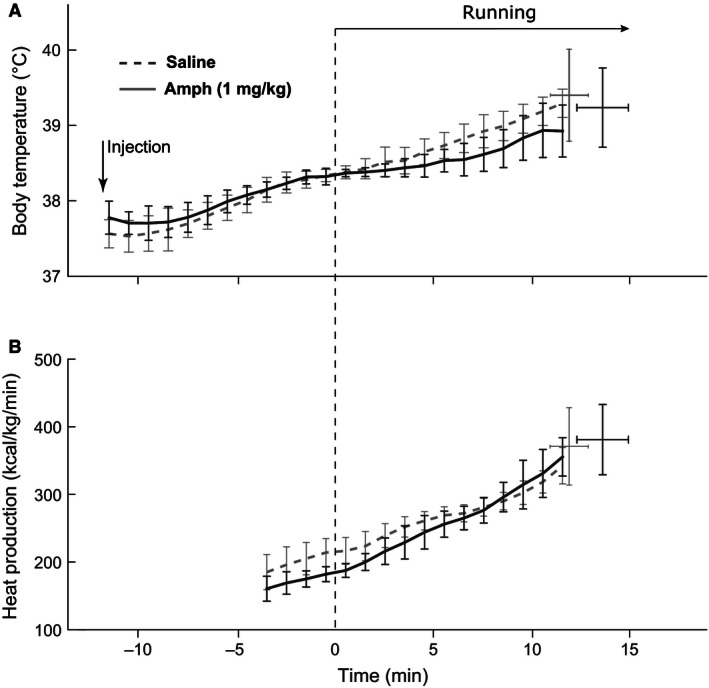
Changes in the core body temperature and heat production in rats running on a treadmill after 1 mg/kg of amphetamine. (A) The body temperature after saline injection (dashed line) or amphetamine injection (solid line). Error bars represent standard deviations over a group of rats. (B) Heat production calculated from O_2_ consumption and CO_2_ production.

**Figure 3:**
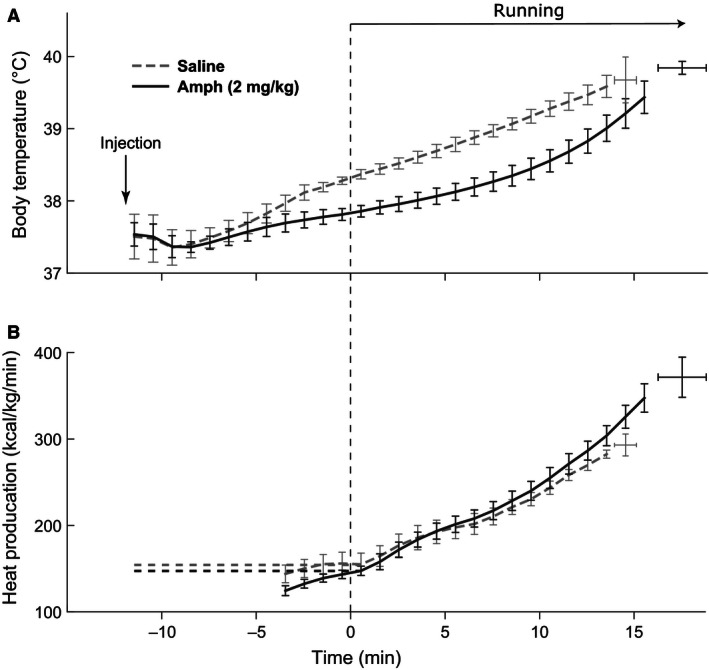
Changes in the core body temperature and heat production in rats running on a treadmill after 2 mg/kg of amphetamine. (A) The body temperature after saline injection (dashed line) or amphetamine injection (solid line). Error bars represent standard deviations over a group of rats. (B) Heat production calculated from O_2_ consumption and CO_2_ production. For modeling purposes, the heat production was assumed to be constant before the start of run (horizontal dashed lines).

Baseline core body temperature of all rats was uniform in the range of 37.5 ± 0.3°C. As soon as rats were placed on a treadmill, their body temperature dropped slightly (Figs. 2 and 3, from −12 min to −10 min,). The presence of a “hypothermic” phase is related to the decrease in core heat production in physiologic “anticipation” of/during exercise, which was described by us earlier (Yoo et al. 2015). Eventually, the body temperatures of rats in all groups started rising.

Low dose of amphetamine (1 mg/kg, i.p.) significantly affected neither body temperature nor oxygen consumption at any time point (Fig. 2). Similarly, there was no effect of this dose on the body temperature at the time of exhaustion. In contrast, higher dose of amphetamine (2 mg/kg) had significant effects on both temperature dynamics and exhaustion time. The amphetamine‐injected group had lower core body temperature than the saline group throughout the experiment (Fig. 3A). Amphetamine also significantly changed the dynamics of temperature; in rats treated with saline the temperature increased at a steady rate, while temperature increase in the amphetamine group was significantly slower in the beginning of the run (Fig. 3A). Compared to controls, amphetamine extended the time to exhaustion: 17.3 ± 0.6 min versus 14.8 ± 0.8 (*P* < 0.05). Interestingly, the temperature at exhaustion was not significantly different between the groups (40.0 ± 0.2 after amphetamine vs. 39.9 ± 0.2 after saline, *P* > 0.05).

The effect of amphetamine on body temperature during running had no correlation with *V*O_2_ consumption. For all matching workloads there were no significant difference in *V*O_2_ consumption between rats which received amphetamine or saline (Fig. 3B). However, longer runs and, hence, higher workloads after amphetamine were accompanied by a higher maximal *V*O_2_ at the end of run (*V*O_2max_).

### Amphetamine increases heat dissipation

**Figure 4:**
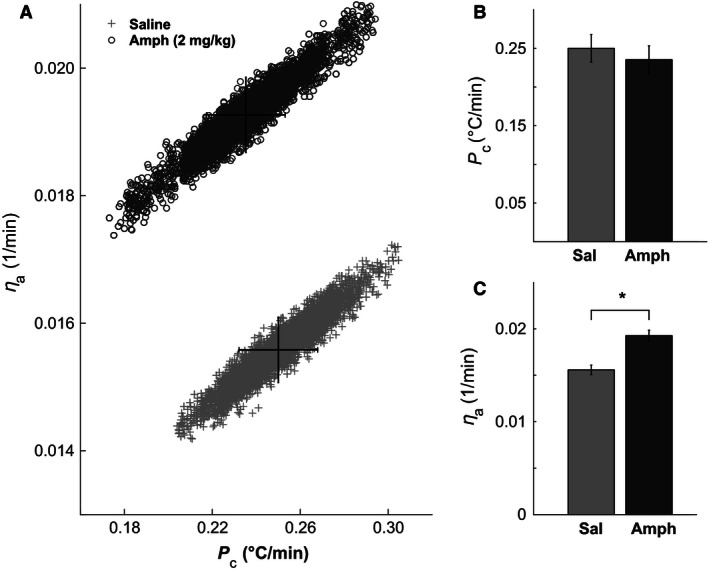
Model parameter estimates. (A) Statistical ensembles of parameters *P*_c_ (heat production in the core) and *η*_a_ (heat exchange between the core and the environment) for saline (crosses) and amphetamine (open circles) generated by Markov Chain Monte Carlo sampling (see text for details). Mean values and standard errors of *P*_c_ (B) and *η*_a_ (C) for saline and amphetamine groups. *Statistically significant difference (*P* < 0.05).

Slower core body temperature increase in the Amph group can be explained either by an increase in heat dissipation, that is, an increase in *η*_a_, or by a decrease in heat accumulation at the core body, that is, a decrease in *P*_c_ (see eq. [1]). We estimated these parameters, *P*_c_ and *η*_a_, by fitting the mathematical model, equations (1) and (2), to the experimental data shown in Figure 3A. Figure 4A contains ensembles of parameters (*P*_c_, *η*_a_) distributed according to the corresponding posterior probability density functions (likelihoods, see Methods). These ensembles were generated by Markov Chain Monte Carlo method as described in the Methods section. Statistical analysis of these samples showed that the heat production in the core body (*P*_c_) was not significantly different between the groups: 0.25 ± 0.02°C/min after saline versus 0.24 ± 0.02°C/min after amphetamine (Fig. 4B). In contrast, the heat dissipation coefficient for the Amph group (0.0194 ± 0.0005 min^−1^) was significantly higher (*P* < 0.005) than heat dissipation in the saline group (0.0156 ± 0.0005 min^−1^) (Fig. 4C).

**Figure 5:**
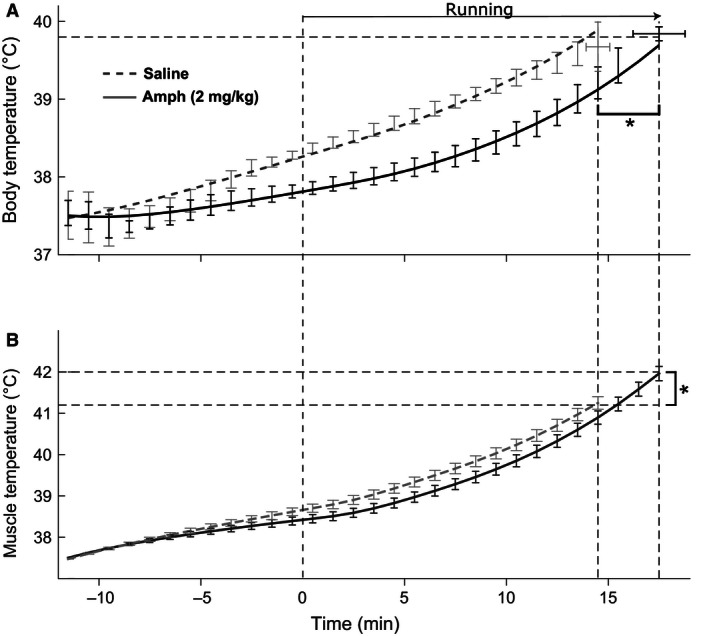
Comparison of experimental data and model performance, and calculated temperature of the muscles. (A) Calculated core body temperature of rats injected with saline (dashed line) or amphetamine (solid line) together with the experimental data (shown by error bars representing the mean ± SD). Dashed vertical lines show the beginning of running (*t* = 0) and the average time of exhaustion in two groups. (B) Calculated muscle temperature dynamics for the two groups. Error bars represent standard errors. *Statistically significant difference (*P* < 0.05).

For each group, we fitted the core body temperature dynamics based on the total heat production in the body (from *V*O_2_), as well as we sampled values of heat production in the core, *P*_c_, and heat dissipation, *η*_a_. As shown in Figure 5A, at most probable values of *P*_c_ and *η*_a_ (best fit), the model reproduced the core temperature dynamics after both saline and amphetamine within one standard deviation.

### Muscle temperature reaches higher levels in amphetamine‐treated rats

Using equation (2), we calculated the heat production by muscles *P*_m_ and then estimated the temperature of the muscles by plugging sampled values of parameters (*P*_c_, *η*_a_), shown on the Figure 4, to equation [Link]. Throughout the run, the muscle temperature in the saline group was slightly higher than in the Amph group. However, the difference in muscle temperature between the two groups was dramatically smaller than the difference of core body temperatures. Due to the fact that after amphetamine treatment rats were able to run almost 3 min longer than control rats, the muscle temperature at the end of the run in the Amph group reached values that were significantly higher than in the saline group. The highest muscle temperatures at the end of run after saline and amphetamine were estimated as 41.2 ± 0.1°C and 41.9 ± 0.2°C, respectively (Fig. 5B).

## Discussion

Amphetamine at low to moderate doses enhances physical performance of humans and animals (Weiss and Laties 1962; Wyndham et al. 1971; Gerald 1978). We observed that relatively low dose of amphetamine (2 mg/kg) increases time to exhaustion in rats exercising with high intensity (high‐speed treadmill running) at room temperature. This dose of amphetamine is similar to those that animals self‐administer (Pickens and Harris 1968; Schenk et al. 2007) and comparable to a typical dose that humans use (~50 mg). Our observations are consistent with previous studies showing enhanced endurance in rodents treated with amphetamine (Bhagat and Wheeler 1973; Molinengo and Orsetti 1976; Gerald 1978). In this study we provide a novel potential mechanistic interpretation of this phenomenon; we show that amphetamine increases heat dissipation which helps the core body temperature to delay reaching the threshold for exhaustion.

Considering that well‐controlled body temperature is one of the determinants of endurance, the goal of the present study was to evaluate the effect of amphetamine on the thermoregulatory system in rats running on a treadmill. We applied mathematical modeling to explain the amphetamine‐induced changes of the body temperature dynamics in running rats. This approach allowed us to calculate parameters that are difficult to measure experimentally, that is, heat dissipation coefficient, heat production in the core and the muscles, and muscle temperature.

Proper functioning of thermoregulatory system plays an important role in ones’ ability to maintain particular levels of physical activity. Excessively high body temperature can cause (or underlie) exhaustion and limit exercise duration. Walters et al. (2000) and Fuller et al. (1998) provided evidence of the existence of a limiting body temperature; they showed that at a point of exhaustion from voluntary running on a treadmill, the abdominal and the hypothalamic temperatures reached certain thresholds. Moreover, the limiting temperatures were independent of ambient (*T*_a_) and initial (*T*_0_) temperatures. In agreement with these experimental observations, the core body temperatures of rats at the end of running due to exhaustion were not statistically different between Amph and saline groups in our study (Fig. 3A). However, at any time point during the run the core body temperature of rats treated with amphetamine remained significantly lower than of control rats. This strongly suggests that amphetamine delayed temperature rise to the critical value at which exhaustion occurred and, as the result, significantly increased the time to exhaustion.

Based on *V*O_2_ and *V*CO_2_ measurements, we calculated the total heat production in both groups. We found no significant difference in heat production during running between the groups, meaning that changes in temperature dynamics are caused either by redistribution of heat production in the body or by an increase in heat dissipation. To discriminate between these two possibilities, we used a mathematical model, equations (1) and (2), describing temperature changes in the core and muscle compartments during run. For this purpose, we adapted the model from Yoo et al. (2015). Previously, for simplicity, heat dissipation to the environment and heat production in the core were combined in a single term, overall net heat production in the core. We augmented that model by incorporating heat production in the core and heat dissipation to the environment explicitly. This improvement allowed us to use experimental data to estimate heat dissipation coefficient (*η*_a_) and heat production parameter $\left(P_{c}\right),$ and then to infer which parameter is affected by amphetamine. We found that amphetamine significantly enhances heat dissipation and has no effect on heat production.

While the rise of *T*_c_ was slowed down by increased heat dissipation, the cooler core was not able to significantly delay the rise of *T*_m_. Therefore, at any selected time the muscle temperature was not significantly different between Amph and Saline groups. However, due to increased running time in Amph group, the muscle temperature in this group was significantly higher at the end of running (see Fig. 5). This difference of almost 1°C brings the temperature of muscles to the level that could result in physical damage of muscle tissue from hyperthermia.

Based on the above, we can hypothesize that exertional exhaustion may be mediated by failure of thermoregulatory mechanisms to keep the core body temperature below the threshold. The same mechanism indirectly allows keeping the temperature of contracting muscles within safe range. Administration of psychostimulants does not change the threshold for the core temperature at room temperature. However, it tricks the thermoregulatory system by an “unauthorized” increase of heat dissipation and, thus, increasing the gap between the temperatures of the core and the muscles, which may be potentially dangerous for the latter.

### Model assumptions

We considered the heat dissipation coefficient *η*_a_ constant throughout the exercise. This assumption is based on the following logic. Major changes of heat dissipation in running rats occur when thermoregulatory mechanisms dilate cutaneous vessels. Tail temperature is a well‐accepted marker of thermoregulatory heat dissipation. Tanaka et al. (1988) measured the body temperature and the tail temperature in rats running with various speeds at room temperature. The threshold temperature for heat dissipation (body temperature at which heat dissipation starts increasing) quickly rises with the increasing work intensity and at the highest workloads it reaches a maximum value of 39.3°C. Therefore, in the beginning of our experimental protocol, the body temperature was too low for cutaneous vasodilation to occur. Later on, the body temperature increased, but so did the workload. As a result, according to Tanaka et al. (1988), the heat loss threshold would not be hit before the core body temperature reached levels of approximately 39°C. In our experiments, the highest *T*_c_ values at the end of the analyzed period were 39.6°C for Saline group and 39.4°C for Amph group. Therefore, it was reasonable to consider that the heat dissipation did not change within the interval used for model calibration.

Also, when cutaneous vasodilation finally kicks in, body temperature in running animals stops rising and in most cases plateaus (Tanaka et al. 1988). In our recordings, we did not observe any instances of temperature plateauing before animals stopped running, which also confirms that the threshold of thermoregulatory changes in heat dissipation was not reached. This allowed us to assume that the heat dissipation coefficient was constant throughout the exercise.

### Amphetamine increases *V*O_2_max by slowing the temperature rise

Our data show that amphetamine per se does not significantly affect oxygen consumption (*V*O_2_) at the same exercise intensity. *V*O_2_max in the amphetamine group is increased because rats injected with amphetamine can run for longer times thus undergoing a higher physical load according to the protocol. The important implication of our study is that due to higher heat dissipation, it takes longer for the temperature of amphetamine‐treated rats to reach the threshold for exhaustion. This is why they continue to run at higher speeds/inclines, and thus, exhibit higher *V*O_2_max at the end of the run. Accordingly, higher *V*O_2_max in the amphetamine group is indirectly caused by higher heat dissipation.

### Amphetamine increases heat dissipation, but does not suppress thermogenesis

Our modeling results show that a relatively low dose of amphetamine (2 mg/kg) increases heat dissipation, which in turn slows down the core body temperature rise. This finding was made possible by simultaneous measurements of the core body temperature and energy expenditure. Core body temperature was significantly lower in the amphetamine group, while energy expenditure was no different between the groups, meaning that total thermogenesis was not affected by amphetamine. Using mathematical modeling we were able to answer the question whether amphetamine redistributes heat generation between body compartments, or it affects heat removal from the body. We found that amphetamine increases heat dissipation from the core, while its effect on heat production is significant neither in the core nor in the muscles. As a direct experimental validation of our result, whole body calorimetry could be used.

Spreading of saliva, which is one of heat dissipation control mechanism in rats, is difficult to employ while running. Water evaporation from the respiratory tract, which is increased during the exercise (Tanaka et al. 1988) due to increased ventilation and higher body temperature is dictated by energy demand rather than thermoregulation. Therefore, rats control their heat exchange with the environment during exercise predominantly by dilating or constricting cutaneous blood vessels. Due to the absence of fur and large surface area, the tail is the major thermoregulatory organ in rats.

More than 40 years ago, Borbely et al. (1974) measured the tail temperature of rats after treatment with various doses of amphetamine. They reported an amphetamine‐induced dose‐dependent decrease in tail temperature, suggesting that amphetamine induces cutaneous vasoconstriction and, thus, decrease in heat dissipation. Considering that hyperthermia induced by amphetamine or its derivatives is one of the major complications and may be life threatening (Fitzgerald and Bronstein 2013; Kiyatkin et al. 2014), an increase in heat dissipation coefficient found in this study is counterintuitive. Nevertheless, using a simple mathematical model, we directly linked heat production (based on oxygen consumption) and body temperature increase (characterizing the retained heat), which allowed for reliable estimation of the heat produced and dissipated. Our estimates unequivocally show a significant increase in heat dissipation coefficient after amphetamine administration and no evidence of altered heat production.

One of possible explanations of the seeming discrepancy is that profound hyperthermia is associated with higher doses (5 mg/kg and above), while we studied relatively low doses (1–2 mg/kg). Our findings may have implications on the mechanistic interpretation of previous experimental results concerned with effects of amphetamine and its derivatives on temperature dynamics. Recently we published experimental and modeling data on extremely complex dose dependence of temperature effects of methamphetamine (Molkov et al. 2014). Our interpretation was that in doses exceeding 1 mg/kg methamphetamine activated an inhibitory component, which reversed effects of excitatory influence of lower doses on heat production. Therefore, the aforementioned intricate dose dependence is a result of delicate balance between activation of excitatory and inhibitory pathways controlling heat generation (Molkov and Zaretsky 2014), while both components are mediated by orexinergic neurotransmission (Behrouzvaziri et al. 2015). In that study, based on existing literature data, we explicitly assumed that at room temperature amphetamines do not increase heat dissipation; an assumption that may require reassessment in light of the findings presented in this study.

We are not aware of any specific mechanisms that can explain the increase of heat dissipation by amphetamine during running. However, we estimated the change of the heat dissipation coefficient to be less than 30%. It is dramatically smaller than more than 10‐fold range of metabolic activity. Assuming that thermoregulatory variations in heat dissipation are comparable in magnitude, and that those variations are in significant part mediated by cutaneous vasodilation, the inhibitory effect of amphetamine on cutaneous vasoconstriction may be modulatory. For example, there is a possibility that activation of alpha2‐adrenoreceptors in ventromedial raphe by amphetamine could be a culprit (Madden et al. 2013), which is similar to what we suggested as a possible mechanisms of inhibitory component in the thermoregulatory effects of methamphetamine (Molkov et al. 2014).

Tail temperature seems to be a good indicator of the changes in heat dissipation mediated by cutaneous vasodilation. However, there are serious limitations on what tail temperature measurements can reveal. Tail flow modulation supports changes of heat dissipation in a multifold range (100‐fold increase of flow), so the change of heat dissipation within 30%, found in our study, will not be statistically significantly reflected in the tail temperature. In addition, other mechanisms should be considered, for example, an increase in respiratory evaporation or a change of insulating properties of fur.

Possible mechanistic interpretations do matter for translational importance of our observations, as some physiological mechanisms in rodents could be missing in humans and vice versa.

### Implications for exercise at different ambient temperatures

An interesting testable prediction provided by our study is that the effect of amphetamine may depend on ambient temperature. Since the heat exchange with the environment is proportional to the temperature gradient (the difference between the body temperature and the ambient temperature), the same change in the heat conductance (heat transfer coefficient in our model) would lead to greater variations in total heat loss in colder environments. Accordingly, we expect that at higher (lower) ambient temperatures the effect of amphetamine on endurance is weaker (stronger) given that the increase in heat conductance is the same.

## Conclusion

Using core body temperature dynamics and mathematical modeling, we estimated parameters that are hard to measure experimentally, that is, the distribution of heat production in the core and muscles and the heat dissipation coefficient. We found that in rats, amphetamine (2 mg/kg) slows down the temperature rise during treadmill exercise at room temperature by increasing heat dissipation. We suggest that this psychostimulant increases the time to exhaustion in rats, at least in part, by delaying the moment when the core body temperature exceeds the threshold defining exhaustion. The calculated muscle temperature at the end of run in rats after amphetamine was almost one degree higher than after saline which may be health threatening. We conclude that while amphetamine improves endurance and extends the time at which exhaustion occurs, its use can result in health‐threatening complications.

## Conflict of Interest

None declared.
